# Draft Genome Sequence of Chilean Antarctic *Pseudomonas* sp. Strain K2I15

**DOI:** 10.1128/genomeA.00771-17

**Published:** 2017-08-17

**Authors:** Paz Orellana, Alequis Pavón, Sandra Céspedes, Lorena Salazar, Ana Gutiérrez, Daniel Castillo, Gino Corsini

**Affiliations:** a Facultad de Ciencias de la Salud, Instituto de Ciencias Biomédicas, Universidad Autónoma de Chile, Santiago, Chile; b BIOREN-UFRO and Departamento de Producción Agropecuaria, Facultad de Ciencias Agropecuarias y Forestales, Universidad de la Frontera, Temuco, Chile; c Marine Biological Section, University of Copenhagen, Helsingør, Denmark

## Abstract

We announce the draft genome sequence of *Pseudomonas* sp. strain K2I15, isolated from the rhizosphere of *Deschampsia antarctica* Desv. The genome sequence had 6,645,031 bp with a G+C content of 60.4%. This genome provides insights into the niche adaptation, prophage carriage, and evolution of this specific Antarctic bacteria.

## GENOME ANNOUNCEMENT

The genus *Pseudomonas* contains the most diverse and ecologically significant group of bacteria on the planet (1). Members of the genus are found in large numbers in all the major natural environments (terrestrial, freshwater, and marine) and are also associated with plants and animals (2). This universal distribution suggests a remarkable degree of physiological and genetic adaptability, which permits a notable capacity of *Pseudomonas* strains to degrade a wide range of substrates and produce antibiotics and plant hormones (3–5). There is no doubt that further acquisition and analysis of whole-genome sequences will reveal substantial information among phenotypic traits of *Pseudomonas* isolates. Here, we report the draft genome sequence of *Pseudomonas* sp*.* strain K2I15, which was isolated from the rhizosphere of *Deschampsia Antarctica* Desv., in the Collins Glacier (62° 22'S, 59° 43'W), localized in the Chilean Antarctic territory.

*Pseudomonas* sp. strain K2I15 was grown overnight at 18°C with agitation in LB broth (catalog no. 12106-05; Mo Bio) Genomic DNA was extracted using the QIAamp DNA minikit (Qiagen) according to the manufacturer’s protocol. A sequencing library was prepared using the Illumina HiSeq platform (MicrobesNG, United Kingdom) with paired-end read sizes of 100 bp. A total of 17,514,936 paired-end reads were used for *de novo* assembly in Geneious version 9.1.6 (6). Short and low-coverage contigs were filtered out, resulting in a set of 140 with an average coverage of 97× (*N*_50_, 94,045 bp). Annotation was performed by using the NCBI Prokaryotic Genome Automatic Annotation Pipeline (PGAAP) (7). Additionally, the genomes were analyzed on the Rapid Annotation using Subsystems Technology (RAST) server (8). Genome island detection was achieved using IslandViewer 4 (9). Acquired antibiotic resistance genes were identified using ResFinder 2.1 (10), virulence factors using VirulenceFinder 1.2 (11), clustered regularly interspaced short palindromic repeat (CRISPR) arrays using CRISPRfinder (12), and prophage-related sequences using PHASTER (13).

The final assembly for *Pseudomonas* sp. strain K2I15 had a total length of 6,645,031 bp and a G+C content of 60.4%. Genome annotation resulted in 5,981 coding sequences (CDSs), 59 tRNAs, 104 pseudogenes, and 7 rRNAs. Screening for genomic islands showed that *Pseudomonas* sp. strain K2I15 harbored fourteen specific genomic islands between 4.5 and 40.4 kb with a G+C content ranging from 45.5 to 53.2%. One intact prophage-like element of 22.2 kb, encoding 22 open reading frames (ORFs) was detected. No virulence genes or CRISPR arrays were identified. Niche adaptation factors were recognized with functions such as copper, arsenic, and zinc tolerance; cold shock CspA-I family of proteins; siderophore production (pyoverdine, achromobactin); and Colicin V and Auxin production. Antibiotic resistance genes to fluoroquinoles, streptothricin, penicillin, and fosfomycin were found. Moreover, we found nineteen multidrug resistance efflux pumps. Thus, this genome sequence can facilitate future comprehensive comparisons, phylogenetic analyses, and niche adaptations of *Pseudomonas* communities.

### Accession number(s).

The draft genome sequence of *Pseudomonas* sp. strain K2I15 can be accessed under the GenBank accession number NIXO00000000. The version described in this paper is the first version, NIXO01000000.
